# Supplementary material for: Energy Drink Consumption, Depression, and Salutogenic Sense of Coherence Among Adolescents and Young Adults
Source: Int J Environ Res Public Health. 2020 Feb 17;17(4):1290. doi: 10.3390/ijerph17041290 (PMC7068601; doi:10.3390/ijerph17041290)
Supplement: Supplementary file 1 [file ijerph-17-01290-s001.pdf]

Beck's Depression Inventory

Instructions: This questionnaire consists of 13 groups of statements. Please read each group of statements carefully. And then pick out the one statement in each group that best describes the way you have been feeling during the past two weeks, including today. Circle the number beside the statement you have picked. If several statements in the group seem to apply equally well, circle the highest number for that group.

B1 Sadness
- 0 I do not feel sad.
- 1 I feel sad.
- 2 I am sad all the time and I can't snap out of it.
- 3 I am so sad and unhappy that I can't stand it.

B2 Pessimism
- 0 I am not particularly discouraged about the future.
- 1 I feel discouraged about the future.
- 2 I feel I have nothing to look forward to.
- 3 I feel the future is hopeless and that things cannot improve.

B3 Past Failure
- 0 I do not feel like a failure.
- 1 I feel I have failed more than the average person.
- 2 As I look back on my life, all I can see is a lot of failures.
- 3 I feel I am a complete failure as a person.

B4 Loss of Pleasure
- 0 I get as much satisfaction out of things as I used to.
- 1 I don't enjoy things the way I used to.
- 2 I don't get real satisfaction out of anything anymore.
- 3 I am dissatisfied or bored with everything.

B5 Guilty Feelings
- 0 I don't feel particularly guilty.
- 1 I feel guilty a good part of the time.
- 2 I feel quite guilty most of the time.
- 3 I feel guilty all of the time.

B6 Punishment Feelings
- 0 I don't feel I am being punished.
- 1 I feel I may be punished.
- 2 I expect to be punished.
- 3 I feel I am being punished.

B7 Suicidal Thoughts or Wishes
- 0 I don't have any thoughts of killing myself.
- 1 I have thoughts of killing myself, but I would not carry them out.
- 2 I would like to kill myself.
- 3 I would kill myself if I had the chance.

B8 Loss of Interest
- 0 I have not lost interest in other people.
- 1 I am less interested in other people than I used to be.
- 2 I have lost most of my interest in other people.
- 3 I have lost all of my interest in other people.

B9 Indecisiveness
- 0 I make decisions about as well as I ever could.

1 I put off making decisions more than I used to.
2 I have greater difficulty in making decisions more than I used to.
3 I can't make decisions at all anymore.

B10 Change in body image
0 I don't feel that I look any worse than I used to.
1 I am worried that I am looking old or unattractive.
2 I feel there are permanent changes in my appearance that make me look unattractive
3 I believe that I look ugly.

B11 Loss of energy
0 I can work about as well as before.
1 It takes an extra effort to get started at doing something.
2 I have to push myself very hard to do anything.
3 I can't do any work at all.

B12 Tiredness or Fatigue
0 I have as much energy as ever.
1 I have less energy than I used to have.
2 I don't have enough energy to do very much.
3 I don't have enough energy to do anything.

B13 Changes in Appetite
0 My appetite is no worse than usual.
1 My appetite is not as good as it used to be.
2 My appetite is much worse now.
3 I have no appetite at all anymore.
